# Targeting the Hypoxic and Acidic Tumor Microenvironment with pH-Sensitive Peptides

**DOI:** 10.3390/cells10030541

**Published:** 2021-03-04

**Authors:** Nayanthara U. Dharmaratne, Alanna R. Kaplan, Peter M. Glazer

**Affiliations:** 1 Department of Therapeutic Radiology, Yale University, New Haven, CT 06520, USA; peter.glazer@yale.edu; 2 Department of Genetics, Yale University, New Haven, CT 06520, USA

**Keywords:** hypoxia, acidity, pHLIP, tumor targeting, peptide nucleic acids, toxins, nanoparticles, imaging

## Abstract

The delivery of cancer therapeutics can be limited by pharmacological issues such as poor bioavailability and high toxicity to healthy tissue. pH-low insertion peptides (pHLIPs) represent a promising tool to overcome these limitations. pHLIPs allow for the selective delivery of agents to tumors on the basis of pH, taking advantage of the acidity of the hypoxic tumor microenvironment. This review article highlights the various applications in which pHLIPs have been utilized for targeting and treating diseases in hypoxic environments, including delivery of small molecule inhibitors, toxins, nucleic acid analogs, fluorescent dyes, and nanoparticles.

## 1. Introduction

Toxicity to healthy tissue can limit the dose and timing of several therapeutics especially cancer therapeutics, impacting quality of life and causing delays in treatments. Therefore, the development of tumor-specific agents would represent an important step towards overcoming these limitations. To date, much of the effort to develop targeted agents with relative tumor specificity has relied on the surface expression of tumor-specific antigens or cancer-specific dependence on signaling pathways. While these approaches have all led to important and meaningful improvements in cancer therapy, by nature of their design they are limited to specific cancer types and subtypes.

pH-low insertion peptides (pHLIPs) represent a potential system to specifically target tumors, and importantly do not rely on the surface expression of tumor-specific antigens. Instead, pHLIPs are a pH-based delivery system that take advantage of the acidic condition of the tumor microenvironment for selective delivery of cargo. Acidity of tumor environments arise mainly as a consequence of hypoxia. As acidity is a ubiquitous hallmark of the tumor microenvironment, pHLIPs have potential for use across a number of different tumor types. This review article outlines pHLIP function and describes the numerous ways that pHLIP has been used in experimental models to demonstrate efficacy for cancer therapy and/or imaging.

## 2. Targeting of the Tumor Microenvironment with pHLIP

In normal tissue, extracellular pH is tightly regulated to approximately 7.4, while in pathological states, such as ischemia, inflammation, and neoplasia, dysregulation of pH commonly occurs. The tumor microenvironment is largely acidic [1], likely as a result of anaerobic glycolysis and lactic acid production by tumor cells. While this is in part due to metabolic shifts in response to tumor hypoxia (known as the Pasteur effect), tumors also show a preference for anaerobic glycolysis even in the presence of oxygen, a phenomenon known as the Warburg effect. Therefore, acidity is a ubiquitous aspect of the tumor microenvironment, and delivery systems targeting low extracellular pH would allow for relatively selective delivery of cargo to the tumors in vivo [2,3].

pH-low insertion peptides (pHLIPs) demonstrate pH-dependent ability to insert across cell membranes [4] and are therefore a promising system for delivery of cancer therapeutic and/or diagnostic agents. pHLIPs are short peptides that are largely unstructured and weakly interact with cell membranes at neutral pH [5]. At acidic pH, pHLIPs adopt helical conformations due to protonation of acidic amino acid residues in the transmembrane domain of the peptide [4]. This pH-dependent conformational change allows pHLIPs to insert their C-terminus across cell membranes only at extracellular pH < 6.5 [4].

**Figure 1:**
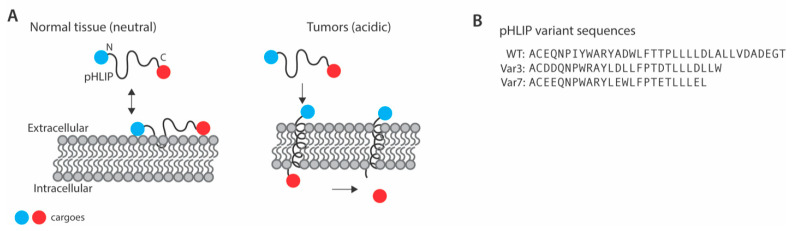
pH-low insertion peptide (pHLIP) mechanism of action. (**A**) Mechanism of action for tumor-specific delivery of cargo or labeling of cell membranes using pHLIP. (**B**) Amino acid sequences of pHLIP and selected pHLIP variants.

Cargo can be linked to the C-terminus of pHLIP using a disulfide bond or other linkers that are subject to cleavage in the intracellular reducing environment [4]. Linkage of cargo to the C-terminus of pHLIP therefore allows for intracellular delivery, whereas linkage of cargo to the N-terminus allows for labeling of tumor cells [6], which may have important implications for use in cancer imaging. Different variants of pHLIP have been developed by changing the amino acid sequence and chain length to fine tune cell uptake (Figure 1). As described in this review, a number of cargoes have been linked to pHLIP for potential therapeutic and/or diagnostic purposes including toxins, small molecules, peptide nucleic acids, dyes, and nanoparticles.

Importantly, the mechanism of action of pHLIP confers three advantageous properties for potential cancer therapeutics. First, the pH-specificity of pHLIP confers relative selectivity in targeting tumor cells, which allows for sparing of normal tissue and therefore an expected reduction in dose-limiting toxicities. Furthermore, the pHLIP system can assist in the transport of otherwise cell-impermeable molecules across the cell membrane, ameliorating potential issues in bioavailability. Finally, pHLIP targets the tumor microenvironment on the basis of acidity, a ubiquitous feature of the tumor microenvironment, thereby distinguishing it from other attempts to develop tumor-specific reagents.

## 3. Biodistribution of pHLIP

**Figure 2:**
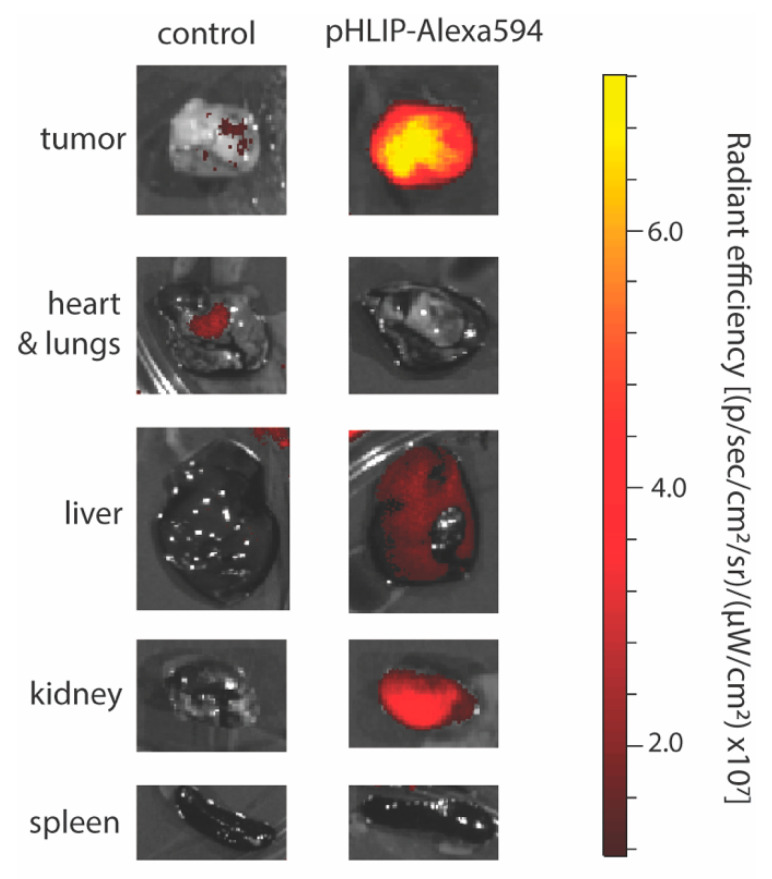
Selective targeting of the tumor microenvironment with pHLIP. Fluorescence in isolated HeLa tumors and selected organs from mice 24 h after systemic (intravenous) administration of vehicle (control) or pHLIP-Alexa594 (2 mg/kg).

In mouse models, fluorescently labeled pHLIP has shown targeting to subcutaneously injected and spontaneous tumors, as well as metastatic lesions (Figure 2) [6]. Accumulation of fluorescent pHLIP in tumors is detected as early as 4 h and persists for at least 72 h [6]. pHLIP can target to small tumors before they are visible by eye [7]. The size of the tumor impacts pHLIP distribution such that in smaller tumors pHLIP distributes evenly, whereas in larger tumors pHLIP has even higher accumulation in necrotic cores [6].

pHLIP shows enhanced targeting to aggressive tumors, as evidenced by the enhanced uptake of fluorescently labeled pHLIP by tumors formed from M4A4 cells, a highly aggressive and metastatic melanoma line, as compared with tumors formed from NM2C5 cells, a less acidic and weakly metastatic melanoma cell line [6]. The ability of pHLIP to target aggressive tumors particularly well can be explained by the relationship between tumor aggressiveness and extracellular acidity [8]. However, pHLIP still demonstrates the ability to target to slow-growing tumors [7].

Many cancer therapeutics are limited by their inability to cross the blood–brain barrier, limiting potential treatment options for brain cancers and metastases. Biodistribution experiments in orthotopic and subcutaneous models of glioma show accumulation of pHLIP is limited to extracranial sites, suggesting that pHLIP is unable to cross an intact blood–brain barrier [9]. However, significant disruption of the blood–brain barrier can occur in aggressive brain tumors such as glioblastoma [10], raising the possibility that pHLIP may be capable of delivering cargo in these tumor types.

Beyond the tumor microenvironment, a number of other pathological states involve the dysregulation of tissue pH, including inflammation [7]. In addition to accumulation at sites of pathologic extracellular acidity, fluorescently labelled pHLIP has also shown accumulation in the kidney, predominantly in the distal cortex, in mouse models [6]. This has been attributed to the role of the kidney in metabolizing low molecular weight proteins such as pHLIP, as well as the acidity found in specific regions of the kidney. Importantly, renal accumulation of pHLIP in mice can be significantly reduced by alkalinization of drinking water to a pH of 8.2 with a bicarbonate buffer [7].

## 4. pHLIP Variants

A number of pHLIP variant sequences have been developed in the effort to improve intracellular delivery as well as specificity for extracellular acidity. Change of either one or two amino acids in the transmembrane region of pHLIP can change the pH_50_ (pH at which 50% of pHLIP are in the inserted state) of pHLIP to improve cell uptake. pHLIP-D25E-C (Asp25 is substituted with Glu), pHLIP-P20G-C (Pro substituted with Gly) have higher pH_50_, whereas pHLIP-R11Q-C (Arg is substituted with Gln) exhibit lower pH_50_ [11]. A number of variant sequences have been designed based on knowledge of the molecular mechanisms of pHLIP function, such as Var3, a slightly truncated variant containing Asp residues, and Var7, which is also truncated and contains Glu residues (Figure 1) [12]. Recently, pHLIP variants using non-canonical amino acids have also been developed. These include the use of γ-carboxyglutamic acid (Gla), which contains two carboxyl groups, and α-aminoadipic acid (Aad), a less polar version of glutamic acid [13]. Polyethylene glycol (PEG) spacers have been used to link 2 or 4 wildtype pHLIP sequences together into so-called “pHLIP bundles” [14]. A rationally designed peptide, acid-triggered rational membrane (ATRAM), has also been developed based in part on the properties of pHLIP [15]. 

The relative efficiency of intracellular delivery and tumor specificity of these pHLIP variants differs on the basis of polarity and size. Truncated variants have reduced membrane affinity at neutral pH due to a loss of hydrophobic residues [12], allowing for potentially enhanced specificity of delivery on the basis of pH. Furthermore, truncation of the pHLIP peptide at the C-terminus, where membrane insertion occurs, confers faster membrane insertion [12]. Across otherwise similar variants, the pKa of membrane insertion is higher in variants containing Glu residues as compared with Asp residues [12]. In vivo, Var3 has high uptake in tumors and good specificity for tumors as compared with uptake in muscle, kidney, and liver [12]. Var7 shows rapid tumor targeting and clearance, which may make it suitable for imaging purposes [12]. Less polar pHLIP variants show more targeting of normal tissue and increased hepatic clearance [14]. “pHLIP bundles” exhibited great efficiency in delivering polar molecules intracellularly in vitro [14]. Therefore, different pHLIP variants may be most advantageous depending on the intended application.

## 5. pHLIP as a Small-Molecule Transporter of Toxins and Drugs to Tumor Cells

Small polar molecules show poor cell uptake in contrast to small hydrophobic molecules due to their hydrophilicity and inability to navigate the lipid bilayer as described by the Lipinski rules of five [16]. Although the cell membrane permeability of these polar drugs and toxins can be facilitated by converting them to non-polar hydrophobic analogs, such complex synthetic approaches may result in loss of their potency. On the other hand, therapeutic agents that are small and hydrophobic may be taken up by all cells with no discrimination, which may be detrimental to healthy cells and decrease the effective concentration in tumor cells [17,18,19,20]. Coupling the inserting end of pHLIP via a disulfide bond or other linker to small polar or non-polar toxins is an effective method of delivering to cells in hypoxic and acidic environments. pHLIP has a unique amino acid sequence that allows transmembrane insertion at low pH, thereby facilitating the delivery of small anti-cancer toxins and drugs to tumor cells evading healthy cells [18,19,20]. Although minor modifications are required to conjugate molecules to pHLIP, these extra synthetic steps are compensated by the proficient delivery directly to the cytoplasm preventing endosomal trapping and increasing therapeutic efficacy. 

Phalloidin and α- amanitin are cell membrane-impermeable, polar, cyclic toxins that can function as cell proliferation inhibitors and anti-cancer agents. Despite their anti-cancer properties, they lack utility as therapeutic agents due to lack of tumor cell specificity, and inability to cross the cell membrane when delivered naked [18,19,20]. Simple coupling of these toxins to pHLIP exhibits effective delivery to the cytoplasm and has been reported to demonstrate high efficacy in multiple tumor models (Table 1) [19]. In the case of phalloidin, this approach eliminates sophisticated modifications of the toxin to convert it to non-polar analogs, and instead modifies the inserting end of pHLIP to enable superior insertion [19]. Functionalization of the inserting end of pHLIP with hydrophobic rhodamine confers vastly superior cell uptake of phalloidin as compared to naked pHLIP. A hydrophobic linker between pHLIP and amanitin enhances cell uptake demonstrating various methods of modulating the polarity of the delivery system [20]. 

Targeted delivery to tumor cells has been achieved with drugs such as doxorubicin and clinically validated anti-cancer agent microtubule inhibitor monomethyl auristatin E (MMAE) utilizing pHLIP [11,21,22]. The efficacy of such agents in cancer cells can be limited by the development of multi-drug resistance (MDR), which arises as consequence of increased drug efflux due to the expression of adenosine triphosphate binding cassette transporters in the cytoplasm. For drugs to perform as therapeutic agents, cleavage from pHLIP is required. Ordinarily, the reducing environment of the cytosol is sufficient to facilitate cleavage of pHLIP from its cargo. However, in the case of doxorubicin, pretreatment with GSH ethyl ester was required to improve cleavage from pHLIP and enhance drug release, further demonstrating different avenues to improve drug efficacy without modifying the drug itself [21]. 

## 6. pHLIP as a Transporter of Nanoparticle-Based Encapsulated Therapeutics to Cancer Cells 

Nanoparticle-based drug delivery systems are popular in cancer therapy due to the ease of targeting disease tissues passively as a consequence of the enhanced permeation and retention (EPR) effect and the ability to encapsulate therapeutic payloads and release them at the disease site [26,27,29,32]. However, in some tumors the EPR effect is small or nonexistent, leading to poor targeted delivery. Nano delivery systems could also lead to multidrug resistance due to random diffusion of payloads in tumor cells and entrapment of nanoparticles within endosomes could result in requirement of higher dosages [26].

Coating nanoparticles with multiple pHLIPs is an active method of transporting nanoparticles to tumor cells by promoting membrane deformation. Doxorubicin loaded mesoporous silica nanoparticles conjugated to the C-terminus of pHLIP via a disulfide linkage exhibit efficient and controlled release in several tumor cell lines (Table 1). In this construct pHLIP not only functions as a targeting agent but also as a barrier. pHLIP gates the nanoparticle and induces the release of doxorubicin directly to the cytoplasm after it has been cleaved in the reducing environment of the cytosol [26]. 

In contrast to cytoplasmic delivery by conjugating to the C-terminus (inserting end) of pHLIP, successful cytoplasmic delivery of nanoparticles conjugated to the N-terminus of pHLIP have also been reported. In one such study, N-terminus of pHLIP was conjugated to the cationic Dendrigraft poly L-lysines (DGL) via a malemidyl-u-N-hydroxysuccinimidyl polyethyleneglycol linkage and utilized to deliver plasmid DNA in vitro and in vivo. Delivery of this construct occurs via three modes, where tumor targeting occurs due to EPR effect, pHLIP mediated membrane internalization as a consequence of acidic environment due to hypoxic conditions, and nanoparticle uptake by adsorption mediated endocytosis [29]. Enhanced accumulation of pHLIP conjugated nanocarriers in acidic cells compared to naked nanoparticles indicate efficient tumor cell targeting, where in vivo studies indicate 86% mRNA inhibition with pHLIP conjugated nanoparticles compared to 19% without pHLIP.

Another study on N-terminus conjugated nanoparticles demonstrates successful delivery of PEGylated liposomes containing C6 ceramide to A549 cells. The study reports two cell entry pathways, 50% attributing to direct fusion with cell membrane and the rest via endocytosis. Ninety percent cancer cell apoptosis was observed in the presence of pHLIP coated liposomes compared to 40% in the absence of pHLIP [27]. In a similar study, successful delivery of gramicidin channel encapsulated liposomes to cell membrane and enhanced apoptosis of HeLa, A549 and M4A4 cells in a pH-dependent manner has been reported, showcasing the nature of pHLIP to promote cellular uptake of nanoparticles [28]. 

There is also interest in using pHLIP to enhance tumor targeting of gold nanoparticles. As gold increases the effectiveness of radiation therapy by absorbing radiation at high rates and releasing low-energy electrons locally through the Auger effect [30], the use of pHLIP to localize delivery of gold nanoparticles to tumor cells would allow for enhanced radiation damage and tumor cell death with sparing of normal tissue. Conjugation of gold nanoparticles to the N-terminus of pHLIP using maleimide (gold-pHLIP) has been shown to increase cellular uptake of gold under acidic conditions, with targeting localized particularly to the plasma membrane [30,31]. In vivo, intratumoral injections of gold-pHLIP allowed for highly effective and specific delivery to tumors [30]. Intravenous injections of gold-pHLIP show enhanced tumor targeting as compared with gold nanoparticles, however gold-pHLIP delivers with greater efficacy to liver, kidney, and spleen than to tumors [30]. Spherical nanoparticles coated with both pHLIP and PEG have also been described, and are characterized by high stability in solution [32]. As compared with gold-pHLIP, gold nanoparticles coated with both pHLIP and PEG may also show improved tumoral uptake after intratumoral injections [32]. In vitro, gold-pHLIP treatment has been shown to reduce cell survival after low-dose radiation therapy (1.5 Gray) [31], however its potential efficacy in vivo remains to be determined.

As another approach to tumor targeting of metallic nanoparticles by pHLIP, recent work explored the ability of pHLIP to deliver gadolinium (Gd) nanoparticles which can serve as theranostic agents [33]. MR imaging of tumor-bearing mice showed pHLIP-Gd nanoparticles had a long retention time in the tumor (>9 h), suitable for radiotherapy, and penetrated into the poorly vascularized tumor core. In cell culture studies, intracellular delivery of Gd nanoparticles was seen to enhance the effect of radiation as expected via short-range radiosensitizing photoelectrons and Auger electrons. 

## 7. pHLIP Mediated Delivery of Genetic Material (Peptide Nucleic Acids)

Treating cancer by targeting the genetic core has gained interest in the past decade. microRNAs (miRNAs) are one of the main targets of such treatments, where some miRNA (oncomiRNAs) overexpression signals the onset and proliferation of cancer [34]. One strategy to inhibit miRNAs is the utility of antisense oligonucleotides such as peptide nucleic acids (PNAs). PNAs function as DNA mimics with a charge-neutral peptide backbone composed of functionalized N-(2-aminoethyl)glycine units. This unique pseudopeptide backbone prevents degradation by nucleases and proteases. PNAs stand out among other antisense oligonucleotides due to its high affinity to complementary RNA and DNA and thermal stability [38]. A significant constraint to the use of PNAs, like most other therapies, is the low cell uptake of these hydrophilic constructs, endosomal trapping of these foreign material and reticuloendothelial clearance [39]. 

An interesting approach for targeted PNA delivery to tumor microenvironments is by conjugating PNA to the inserting end of pHLIP. A recent study revealed that the delivery of pHLIP-PNA construct does not appear to be significantly affected by the sequence of PNA and more dependent on the length of PNA [3]. Shorter PNAs (12 or 16 mer) show best uptake in vitro and in vivo, and a 3-fold decrease is seen with 20mer and longer PNAs [3]. Nevertheless, it is essential to note that hydrophobicity of large PNAs determines the degree of cell uptake and not purely the length of the PNA. Factors such as gamma modification of PNA, functional groups, and frequency of modification that alter the hydrophobicity of PNA should be considered when designing PNA-pHLIP constructs for efficient cell uptake [3,34,37].

Recent studies show the successful delivery and efficacy of PNAs conjugated to the C-terminus of pHLIP, where cells under hypoxic acidic conditions promote non endocytic cell uptake and release of PNAs to the cytoplasm by disulfide reduction. Successful delivery and high efficacy of anti-miR155-pHLIP construct was reported in two mouse lymphoma models [34]. Significantly less accumulation in the liver was seen with anti-miR155-pHLIP compared to naked anti-miR155. In comparison to agents used in the standard of care for treatment of human lymphoma (doxorubicin and CHOP (cyclophosphamide, doxorubicin, vincristine, and prednisolone)), anti-miR 155-pHLIP showed equivalent effects in delaying tumor growth and suppressing the metastatic spread. Therapeutic effects of anti-miR 155-pHLIP when compared with commercially available locked nucleic acid (LNA) (Exiqon) used for silencing miRNAs was significantly higher. The dosage of anti-miR155-pHLIP used was multiple fold lower than what has been reported in other miRNA studies, demonstrating the clinical viability and importance of further exploring this delivery system [34,39].

A pHLIP-based approach has also been used to target the oncogenic miRNA miR-21, which plays an essential role in tumor pathology by regulating cancer cell growth, proliferation, and chemotherapeutic resistance [35]. More recently, miR-21 expression in tumor-associated macrophages has been linked to the tumor immune response, such that inhibition or genetic deletion of miR-21 causes an improved anti-tumor immune response [35]. Treatment of tumor bearing mice with a pHLIP-conjugated PNA targeting miR-21 (pHLIP anti-miR21) reduces tumor growth and increases tumor cell death [35]. Interestingly, pHLIP anti-miR21 treatment has similar effects in a model system with genetic miR21 depletion specifically in tumor cells, suggesting that the effects of pHLIP anti-miR-21 treatment can be attributed at least in part to suppression of miR21 signaling in non-cancerous cells within the tumor microenvironment [35]. Indeed, uptake of pHLIP-conjugated miR21 was observed in tumor-associated macrophages, making these cells a potential mediator of the anti-tumor effects of miR-21 suppression [35].

Long non-coding RNA (lncRNA) is another target in cancer treatment. HOX transcript antisense RNA (HOTAIR) is a lncRNA reported to be a therapeutic target in ovarian cancers since it contributes to cellular senescence [40]. Existing therapeutic approaches include using siRNA and LNA to target lncRNA. However, these approached are limited by thermal instability, degradation by ribonuclease and inefficient targeted delivery [36]. Utility of PNAs to target lncRNA is a relatively novel concept, but an approach with great promise. When a PNA-pHLIP construct targeting a single stranded region of the HOTAIR RNA was introduced to mice with platinum-resistant ovarian tumor xenografts, tumor growth reduction was observed [36]. This PNA-pHLIP construct in combination with cytotoxic therapeutics exhibited enhanced tumor suppression indicating the effectiveness and adaptability of pHLIP mediated delivery of PNA to target lncRNA and tumor suppression.

## 8. pHLIP-Based Approaches to Targeting DNA Repair and DNA Damage

Efforts have also been made to use pHLIP to deliver cargo to induce DNA damage or inhibit DNA repair pathways for potential use in cancer therapy. Many existing cancer therapies such as platinum-based chemotherapies and radiation therapy act by inducing DNA damage, which tumor cells must repair to ensure cell survival. Therefore, the inhibition of DNA repair in tumor cells is a promising target to increase the efficacy of these treatments. Agents targeting DNA repair through inhibition of poly (ADP-ribose) polymerase (PARP) inhibitors were recently approved by the FDA, and a number of agents targeting the DNA damage response are currently in clinical trials [41]. However, normal tissues must retain intact DNA repair pathways to manage endogenous levels of DNA damage. The use of DNA damaging agents and DNA repair inhibitors can be limited by toxicities to normal tissue, such as the inflammation and fibrosis induced by radiation therapy and cytopenias induced by PARP inhibitors [42,43]. Therefore, the relative tumor selectivity that pHLIP confers could be used to increase the effectiveness of these therapies without increasing their clinical toxicities. 

Recently, pHLIP has also been used to target DNA repair in tumors as a means of increasing the efficacy of radiation therapy [37]. Cell survival after exposure to ionizing radiation requires DNA repair through the non-homologous end joining (NHEJ) pathway [44,45]. While there is interest in developing small molecule inhibitors targeting the NHEJ pathway [46,47], their therapeutic potential is limited by a lack of specificity and in vivo toxicity [48,49]. Increased sensitivity to radiation therapy has been observed in vitro with antisense-mediated suppression of Ku80 [50,51], a key factor in the NHEJ pathway [52,53] that lacks enzymatic activity and therefore is considered classically “undruggable”. Antisense PNAs targeting Ku80 have been successfully targeted to pHLIP, allowing for relatively selective delivery to tumors, causing improved tumor response to radiation therapy and minimal toxicity [37]. Of note, gamma modified PNAs were used to improve solubility, nucleic acid binding, and antisense activity [37,54].

As previously described, while pHLIP can be used to deliver nucleic acid-based inhibitors of classically “undruggable” targets, there is also interest in using pHLIP to deliver small molecule inhibitors to tumors. Such approaches can be challenging, as they require the development of linker molecules to allow for the conjugation and intracellular delivery of said inhibitors to pHLIP while also retaining their activity against target molecules. Nonetheless, promising developments have been made in the development of a pHLIP-conjugated PARP inhibitor [55,56]. While the clinical use of PARP inhibitors as a monotherapy is mostly in cancers with defects in the homologous recombination pathway of DNA repair [57,58,59,60,61,62,63], PARP inhibitors may have efficacy in DNA repair proficient cancers if used in combination with DNA-damaging chemotherapy. However, to date this approach has been limited by toxicity, primarily to the bone marrow [43]. In preclinical models, pHLIP-conjugated PARP inhibitors have been shown to synergize with DNA damaging agents such as temozolomide and irinotecan, and also have minimal normal tissue toxicity [55,56]. 

A pHLIP-based topoisomerase inhibitor has also been developed [64]. Topoisomerase inhibitors block the action of the topoisomerase enzymes that relieve DNA supercoiling caused by replication, transcription, and chromatin remodeling [65]. These drugs are an essential component of chemotherapy in the treatment of a number of cancers, and the topoisomerase inhibitor irinotecan has shown promising results when used in combination with oxaliplatin and 5-FU in pancreatic cancer [66]. However, the use topoisomerase inhibitors can be limited by their toxicities, which include severe diarrhea and cytopenias. While there is potential to use antibody-drug conjugates to limit toxicity, this approach is limited by its requirement for tumoral expression of specific antigens. Alternatively, pHLIP-based approaches can target solid tumors regardless of antigen expression. Conjugation of the highly potent topoisomerase inhibitor exatecan to pHLIP conferred enhanced tumor targeting and tumor growth suppression with sparing of the bone marrow [64]. 

## 9. pHLIP as a Tool for Diagnosing and Imaging Tumor Microenvironments

Acidity is a feature of tumors of every stage, where lowest pH is seen with most aggressive tumors. Thus, imaging based on targeting low pH environments offers evidence on precise tumor location and progression enabling improved therapeutic outcomes [67]. Near-infrared (NIR) fluorescence imaging provides the ability to visualize small animals (whole body), tissues and organs by passing through them for multiple centimeters. Designing constructs where NIR fluorophores are either directly conjugated to the N-terminus of pHLIP or encapsulated within pHLIP decorated nanoparticles provides tools for labeling and imaging cells exposed to hypoxic and acidic environments. 

One such study reveals the kinetics of pHLIP localization when injected intraperitoneally to mice bearing breast adenocarcinoma, where pHLIP detects tumors within 4 h and complete tumor localization is seen within 20 h post injection, even in tumors that were visually undetectable [7]. A remarkable feature of this construct is the ability for it to uniquely target and distinguish between non-metastatic and aggressively metastatic tumors purely based on extracellular pH. Greater staining was observed in M4A4 metastatic tumor compared to NMM2C5 [6]. pHLIP-fluorophore constructs are also able to stain metastatic lesions thereby providing specific prognosis showcasing the power of this technology [6]. Prominent advances in pHLIP technology have led to the utility of pHLIP-indocyanin green (ICG) construct for the fluorescence guided surgical resection of breast tumors. It has the power to accurately identify tumor margins, tumor stroma, flat lesions and micro metastasis close to primary tumor loci [24]. Unlike small molecule fluorophore technology that washout immediately, pHLIP-fluorophore persistency could be modulated by varying the pHLIP variant used [24]. In the case of var 0 or WT pHLIP, the signal remained for multiple days and was nearly five times greater in tumor microenvironments than in other areas [6,7]. 

In another study, different variants of pHLIP were compared to determine the efficiency of cell uptake and persistency of the label. A 4T1 murine xenograft model that is similar to a stage IV human breast cancer was used. pHLIP-fluorophore administered via tail-vein intravenous route revealed the highest tumor uptake with Var3 (Figure 1), with highest fluorophore contrast seen at 24 h. With Var7 (Figure 1), fast clearance was observed with maximum tumor labeling seen at 4 h. The distribution of pHLIP within tumors was homogenous for small tumors, whereas with large tumors maximum accumulation was seen at the center [6,23].

Multiple mouse tumor models containing transgenic breast, prostate, melanoma, and pancreatic models have been used to demonstrate the fluorophore labeling property of pHLIP. However, an area of significant interest in pHLIP mediated detection is the recognition of urothelial dysplasia. Urothelial dysplasia leads to urothelial carcinoma in most cases and early detection of it could provide better therapeutic effects [68]. However, dysplasia has not been clinically detectable. NIR fluorescence dye ICG conjugated to the N-terminus of pHLIP has been used to identify urothelial carcinoma, and there is evidence that ICG-pHLIP may be able to visualize dysplasia as well, allowing early detection of precancerous lesions [68]. However, further work is needed to more precisely determine the sensitivity of pHLIP to identify dysplastic lesions, and it is unknown if the findings in urothelial dysplasia will translate to dysplasia in other tissue types.

pHLIP has also been utilized for the delivery of radioactive agents to tumor cells. The insertion of pHLIP across the cell membrane and retention of N-terminus conjugated radioactive material on the cell surface improves upon the limitations of positron emission tomography (PET) and single-photon emission computed tomography (SPECT) by improving contrast with the background due to longer retention in tumors [9,25]. Var3 pHLIP constructs showed the best imaging properties when used to deliver ^64^Cu and ^18^F to 4T1 orthotopic tumor-bearing BALB/c mice [9]. These constructs have the potential to be transferred as clinically significant novel nuclear imaging markers for hypoxic tissues.

## 10. Non-Tumor Applications of pHLIP Technology 

In addition to cancer imaging and therapeutics, pHLIP technology has potential for use in pathologic states involving the dysregulation of tissue pH, specifically inflammation and ischemia. In vivo imaging shows localization of a pHLIP-conjugated fluorescent dye to sites of inflammation in a rat model of arthritis induced by the injection of methylated BSA and Freud’s complete adjuvant into the knee joint [7]. In inflamed joints, fluorescent signal was detected at approximately 5 times the level of control joints [7]. 

Acid production during cardiac ischemia is a well-described phenomenon [69], raising potential for pHLIP-based approaches in the diagnosis and management of this condition. pHLIP peptides and pHLIP-coated liposomes accumulate in two mouse models of cardiac ischemia: coronary artery occlusion and low-flow global ischemia [70]. pHLIP accumulation in models of cardiac ischemia has potential for clinical relevance, as it is estimated that pHLIP insertion occurs within the range of extracellular pH that induces ischemic chest pain (due to activation of acid-sensing ion channels in cardiac sensory neurons) [70]. Importantly, pHLIP could be used to detect areas of ischemia prior to the onset of extensive tissue damage and irreversible changes to the myocardium [70]. Therefore, pHLIP may be useful for delivery of therapeutic, diagnostic, or theranostic agents in cardiac ischemia.

As pHLIP also targets to the renal interstitium, it may also have value in delivering compounds with relative selectivity to the kidney. Recently, pHLIP was used to renally deliver a PNA targeting miR-33, a microRNA that plays a key role in inflammation, in a mouse model of kidney injury induced by the administration of folinic acid [71]. This anti-miRNA treatment using pHLIP helped reduce the development of renal fibrosis, providing evidence of the utility for pHLIP in treating kidney pathologies [71]. 

## 11. Limitations of pHLIP Technology

A major limitation of pHLIP technology arises due to the synthetic inefficiency of pHLIP-disulfide (SS)-cargo construct. Although the synthesis is straightforward, homodimers of either pHLIP-SS-pHLIP or cargo-SS-cargo, significantly decreases the yields of pHLIP-SS-cargo constructs. This imposes a synthetic burden, reducing the cost effectiveness of the technology and therefore requiring further optimization [3,9].

Simple conjugation of pHLIP facilitates the translocation of most cell impermeable cargos into the cell. However, limitations to delivery of these cargos arise as a result of polarity and size of cargos. Some small polar molecules require further modification of the cargo or the inserting end of pHLIP to enable the effective delivery to the cytoplasm [18,19]. Furthermore, in the case of large polar molecules like PNAs, the size of the cargo dramatically alters uptake [3]. Effective pH-targeted delivery is therefore not only dependent on pHLIP, but also on the cargo, thus pHLIP may not be an effective vehicle for all cargos.

Another limitation of the use of pHLIP for cancer imaging and diagnostic purposes in vivo is the accumulation of pHLIP in the kidneys and in pathological states such as inflammation. While pHLIP still confers a relative tumor-specificity, further optimization of pHLIP technology may allow for improvements in its tumor selectivity. Alternatively, clinical use of pHLIP-based technologies may require the screening of patients for the absence of inflammatory disorders.

Despite these limitations, there is much interest in the clinical use of pHLIP. A phase 1 clinical trial is currently ongoing to evaluate the safety of a pHLIP-based imaging agent ([18F]AlF-cysVar3 pHLIP^®^, NCT04054986). Furthermore, pHLIP-conjugated exatecan is expected to enter clinical trials soon based on promising preclinical data.

## 12. Conclusions and Perspectives

Acidity is a characteristic common to tumors that occurs partly due to metabolic alterations in response to hypoxic microenvironments. In animal models, exploiting acidity for targeted tumor delivery has been achieved using pHLIP for therapeutic and imaging purposes. Conjugating molecules to the inserting end allows cytoplasmic delivery, whereas labeling of acidic cells can be achieved by conjugating to the N-terminus. Multiple variants of pHLIP with altered pharmacokinetic properties have been reported that can be manipulated for various applications. pHLIP precisely delivers small polar toxins, DNA repair inhibitors, imaging agents, nanoparticles and antisense peptide nucleic acids to acidic environments in vivo. PNAs stand out among the numerous cargos delivered by pHLIP since they are non-toxic, sequence specific DNA mimics that can regulate gene expression by targeting microRNAs and mRNAs and can be effective against oncologic targets that are otherwise undruggable. The use of pHLIPs to deliver highly potent small molecule toxins to tumors may also provide advantages over antibody-drug conjugates for the same purpose because pHLIP targeting does not depend on expression of a specific antigen but instead exploits the acidity of solid tumors.

While pHLIP remains a promising technology, improving the synthetic efficiency of pHLIP cargo constructs could significantly facilitate its clinical translation. In recent years, the discovery of multiple variants of pHLIP and new conjugation approaches have expanded the possibilities of cargoes that pHLIP can deliver to the acidic and hypoxic tumor microenvironment. Ultimately, this raises new potential for the use of pHLIP in the treatment and diagnosis of cancer.

## Figures and Tables

**Table 1:** Selected cargo types delivered by pH-low insertion peptides (pHLIP).

Cargo Type	Cargo	Tumor Model	Vehicle
Small Molecules			
***Antiproliferative agents***	Phallodin [19]	HeLa, JC, M4A4, HT1080 *	WT pHLIP-K(rho)
	α-Amanitin [20]	HeLa, U2OS, M4A4, MDA-MB-231 *	WT pHLIP
	Doxorubicin [21]	MCF-7, MCF-7/ADR *	WT pHLIP
	microtubule inhibitor monomethyl auristatin E (MMAE) [22]	HeLa, A431, MDA-MB-23 * NCRr nu/nu mice bearing HeLa or A431 tumors **	WT pHLIP pHLIP-D25E pHLIP-P20G pHLIP-D14Gla:D25Aad
	Paclitaxel [13]	A549 *	WT pHLIP pHLIP-D14Gla:D25Aad
***Imaging agents***	Alexa750, Cy5.5 [6,7]	BALB/c nude mice bearing HeLa and NM2C5 tumors, TRAMP mice bearing M4A4 tumors ** CRL-2116 tumor bearing C3D2F1 mice **	WT pHLIP
	Alexa488, Alexa546, Alexa647, IR680 [23]	4T1 * BALB/c mice bearing 4T1 tumors and MMTV-PyMT mice **	WT pHLIP Var3 pHLIP Var7 pHLIP
	Indocyanin green (ICG) [24]	HMEpC * BALB/c mice bearing 4T1, MDA-MB-231, A549, LLC, M4A4, HeLa, UM-UC3 and LNCaP tumors **	Var3 pHLIP
	^64^Cu, ^18^F-AlF [9]	BALB/c mice bearing 4T1 tumors **	WT pHLIP Var3 pHLIP Var7 pHLIP
	^99m^TC [25]	LLC tumor bearing C57BL/6 mice, PC-3 and LNCaP tumor bearing SCID mice **	WT pHLIP
**Nanoparticles**			
***Antiproliferative agents***	Doxorubicin encapsulated MCM-41 nanoparticles [26]	MCF-7, MCF-7/ADR *	WT pHLIP
	Ceramide encapsulated liposomes [27]	A549, HeLa *	WT pHLIP
	Gramicidin A encapsulated liposomes [28]	A549, HeLa, M4A4 *	WT pHLIP
***Genetic material***	Plasmid DNA encapsulated dendrigraft poly lysine nanoparticles [29]	Bel-7402 * BALB/c nude mice bearing Bel-7402 tumors **	WT pHLIP
***Metallic nanoparticles***	Gold nanoparticles [30,31,32]	HeLa, A549, JC BALB/c nude mice bearing HeLa tumors and BALB/c mice bearing JC tumors **	WT pHLIP
	Gadolinium nanoparticles [33]	A549 * EMT6 tumors in BALB/c mice **	WT pHLIP
**Peptide Nucleic Acids (PNAs)**			
***Anti-miRNA***	Anti-miR-155 [34]	A549, KB, DLBCL * Nude mice bearing neoplastic B cells derived from enlarged spleens of miR-155*^LSLtTA^* mice and mice bearing KB tumors **	WT pHLIP
	Anti-miR-21 [34,35]	A549 * Orthotopic and heterotopic LLC tumors in C57BL6 mice **	WT pHLIP Var3 pHLIP
***Anti-lncRNA***	HOX transcript antisense RNA targeting PNA [36]	A2780P, A2780_CR5, KURAMOCHI, SKBR-3, MCF-7, MDA-MB-231 * BALB/c-nu/nu mice bearing A2780_CR5 tumors **	WT pHLIP
***Antisense***	αKU80 [37]	A549, DLD1-BRCA2KO, EMT-6 * BALBc/Rw mice bearing EMT-6 tumors, athymic nu/nu mice bearing DLD1-BRCA2KO tumors **	WT pHLIP

* In vitro studies, ** In vivo studies.

## Data Availability

This is a review article.
